# The Perception of Cooperativeness Without Any Visual or Auditory Communication

**DOI:** 10.1177/2041669515619508

**Published:** 2015-12-09

**Authors:** Dong-Seon Chang, Franziska Burger, Heinrich H Bülthoff, Stephan de la Rosa

**Affiliations:** Department of Human Perception, Cognition and Action, Max Planck Institute for Biological Cybernetics, Tübingen, Germany; Graduate School of Neural & Behavioural Sciences, International Max Planck Research School, University of Tübingen, Tübingen, Germany; Department of Human Perception, Cognition and Action, Max Planck Institute for Biological Cybernetics, Tübingen, Germany; Department of Human Perception, Cognition and Action, Max Planck Institute for Biological Cybernetics, Tübingen, Germany; Department of Brain and Cognitive Engineering, Korea University, Seoul, Korea; Department of Human Perception, Cognition and Action, Max Planck Institute for Biological Cybernetics, Tübingen, Germany

**Keywords:** Action, perception, cooperation, coordination, social interaction, reciprocity

## Abstract

Perceiving social information such as the cooperativeness of another person is an important part of human interaction. But can people perceive the cooperativeness of others even without any visual or auditory information? In a novel experimental setup, we connected two people with a rope and made them accomplish a point-collecting task together while they could not see or hear each other. We observed a consistently emerging turn-taking behavior in the interactions and installed a confederate in a subsequent experiment who either minimized or maximized this behavior. Participants experienced this only through the haptic force-feedback of the rope and made evaluations about the confederate after each interaction. We found that perception of cooperativeness was significantly affected only by the manipulation of this turn-taking behavior. Gender- and size-related judgments also significantly differed. Our results suggest that people can perceive social information such as the cooperativeness of other people even in situations where possibilities for communication are minimal.

Perceiving social information of an interaction partner is crucial for human interaction (Frith & Frith, 2007; Schilbach et al., 2013). Especially, being able to perceive whether another interaction partner is cooperative or competitive seems to be important for effective real-time coordination with interaction partners (Pfeiffer, Timmermans, Bente, Vogeley, & Schilbach, 2011; Streuber, Sebanz, Knoblich, Bülthoff, & de la Rosa, 2011). Previous studies have also shown how nonverbal forms of communication such as eye gaze, gestures, or kinematics play an important role for successful interactions (Kuhlen, Galati, & Brennan, 2012; Pezzulo, Donnarumma, & Dindo, 2013; Pfeiffer et al., 2011). However, studies investigating real-time interaction without the exchange of social information conveyed by visual or auditory channels have been rare, and we wanted to test this in a situation where possibilities for communication were minimalistic.

How do people perceive the cooperativeness of others if possibilities to exchange visual or auditory information are limited during real-time interactions? Theories of human cooperation would predict that humans have clear beliefs and expectations about the behavior of others, so that individuals complying with these expectations would be seen as more cooperative (Fehr & Fischbacher, 2004; Tomasello, Carpenter, Call, Behne, & Moll, 2005). Humans also seem to have a default expectation of reciprocity and judge the cooperativeness of others based on this presumed disposition of interaction partners (Pfeiffer et al., 2011). The same theories would also predict that humans make person-related inferences about interaction partners even when not much information is present, since inferring the intentions of a partner is natural to almost any human interaction.

In the present study, we connected two participants with a rope, and they had to accomplish a joint task together. The task consisted of collecting points on a laptop computer which was located on each side and could only be reached if both interaction partners coordinated their movements (Figure 1). Importantly, participants were separated by a visual barrier and used a noise-cancelling headphone so they could not see or hear each other in order to make direct communication impossible. The only source of information about the other partner was the haptic force-feedback conveyed through the rope during the simultaneous joint coordination. During the first pilot trials (4 dyads, *n* = 8), we observed a consistently emerging reciprocal “turn-taking behavior.” Although multiple coordination patterns were possible, it seemed as if participants were automatically expecting their partners to collect their points alternately and take turns. We wanted to test whether this turn-taking behavior was associated with perception of cooperativeness and installed a confederate who either maximized or minimized this behavior. Furthermore, do participants make person-related inferences about their interaction partner even when possibilities for communication are minimalistic?

**Figure 1.**
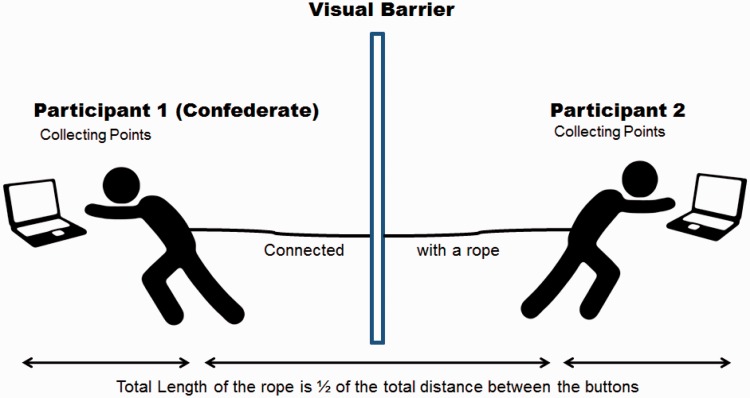
Joint Task: A dyadic pair had to collect points together by pressing eight buttons (a–h) in the right order on a laptop located on each side. All participants were separated by a visual barrier. Points were paid out as money proportional to the collected number of points, but only if both participants successfully finished collecting the specified number of points.

We tested 24 participants (*f* = 12, *m* = 12) who interacted with the same confederate in two sessions while they believed that they interacted with different partners in the two sessions. The confederate maximized “turn-taking” (being fast and predictive with alternating the collection of points) in one and minimized “turn-taking” (being slow and reluctant to alternating the collection of points) in the other session. The order of the sessions was balanced. After each session, participants rated the cooperativeness of the interaction partner on a Likert scale (1–7) and gave estimates about person-related attributes such as the gender and size of their interaction partners.

The results showed that perceived cooperativeness of the interaction partner significantly differed (*t*(23) = −7.11, *p* < .001) depending on whether the interaction partner was engaging in “turn-taking behavior” (*M* = 6.38, *SD* = 1.28) or not (*M* = 3.63, *SD* = 1.47; Figure 2).

**Figure 2.**
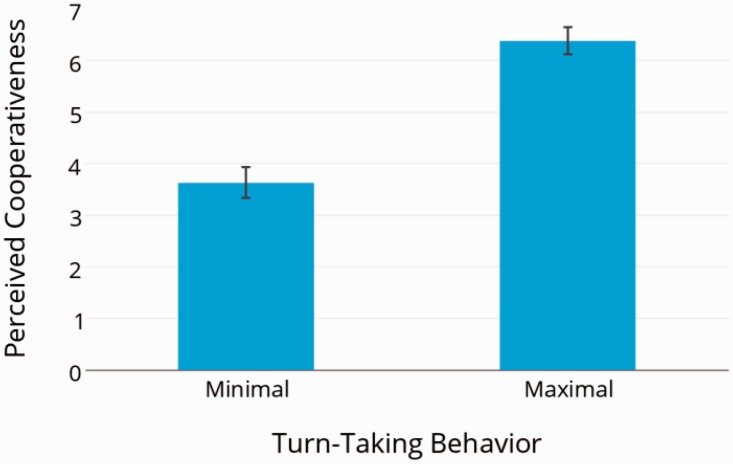
Perceived cooperativeness: We compared minimizing or maximizing the “turn-taking behavior” of the interaction partner. Error bars are depicted as standard error of the mean.

Moreover, participants made different person-related inferences about the gender and size of the confederate (Figure 3). Cooperative partners were more often perceived as female, whereas less cooperative partners were more often perceived as male (*t*(41) = −3.53, *p* < .01). The perceived body size of interaction partners was also significantly different dependent on the partner’s behavior (*t*(24) = 1.29, *p* < .001).

**Figure 3.**
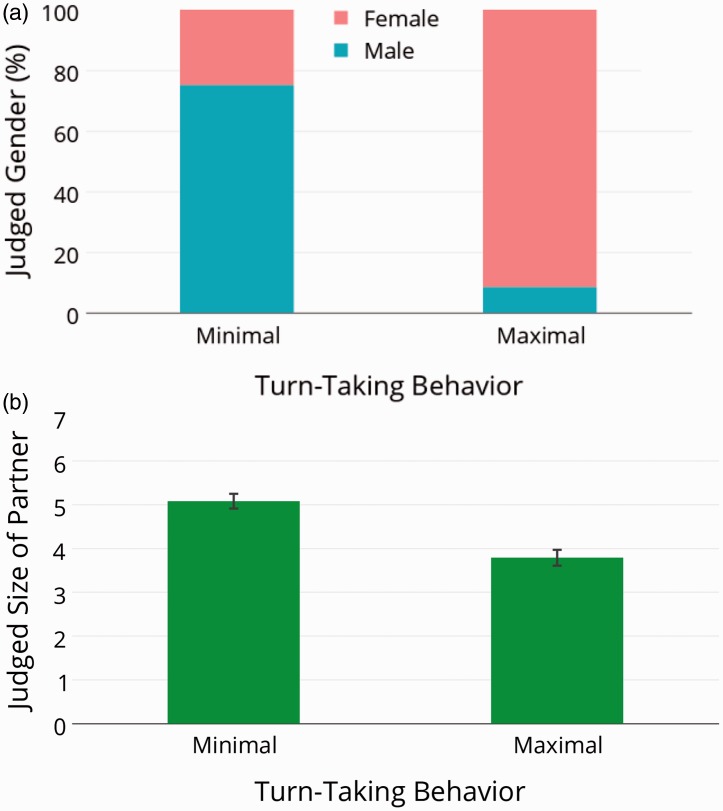
Person-related inferences: (a) Judged gender of the interaction partner. (b) Judged size of the interaction partner. Error bars are depicted as standard error of the mean.

In summary, our results suggest that people collect social information and make inferences about their interaction partner even when possibilities for communication are minimalistic. It is noteworthy that a reciprocal “turn-taking behavior” automatically emerged in the interactions and manipulating this behavior influenced the perception of cooperativeness as well as person-related inferences of the partner. Furthermore, our experimental paradigm of directly connecting two individuals and only allowing haptic force-feedback could serve as a useful method to further study how joint motor coordination is coupled to the perception of social information.
